# EOLA1 functions in nucleotide salvage through deacetylating free N4-acetylcytidine

**DOI:** 10.64898/2026.08.01.741249

**Published:** 2026-08-04

**Authors:** Sebastien Relier, Sarah Schiffers, Hamid Beiki, Maria Prigge, Nishu Tyagi, Cyrinne Achour, Ayush Raman, A. Maxwell Burroughs, L. Aravind, Shalini Oberdoerffer

## Abstract

RNA-based medicines rely on modified nucleotides to promote immune evasion and in vivo efficacy. Nucleotides generated from RNA degradation are either exported or recycled through metabolically favorable salvage pathways, though whether modified nucleotides are efficiently recycled remains unclear. N ^4^ -acetylcytidine (ac⁴C) is a naturally occurring modification in rRNA and tRNA that has shown promise in therapeutic mRNA applications. However, N ^4^ -acetylation impairs cytidine deamination, the first step in cytidine salvage. Here, we investigate the endogenous mechanisms that enable ac⁴C metabolism. Through sensitive sequence and structural analyses, we identify the uncharacterized human ASCH domain protein EOLA1 as a key ac⁴C deacetylase in nucleotide salvage. EOLA1 inactivation leads to free intracellular ac⁴C accumulation and increased cytotoxicity upon nucleotide export inhibition. While steady-state ac⁴C levels in cellular RNAs remain unchanged, EOLA1-dependent regulation of free ac⁴C is evident basally and is exacerbated by exogenous mRNA delivery. Proteomic analyses place EOLA1 in proximity to ribosomal proteins, adjacent to endogenous ac⁴C sources. In vitro assays confirm EOLA1 specificity for ac⁴C, and structural analysis reveals a narrow nucleotide-binding pocket consistent with mononucleotide selectivity. These findings identify EOLA1 as a bona fide ac⁴C eraser and uncover a previously unrecognized pathway for recycling modified nucleotides with relevance to therapeutic RNA design.

## Full Text Availability

The license terms selected by the author(s) for this preprint version do not permit archiving in PMC. The full text is available from the preprint server.

